# You Are The One Who Was Beating Me": A Case Report of a Patient with Postictal Psychosis Secondary to Bilateral Temporal Lobe Epilepsy

**DOI:** 10.7759/cureus.6069

**Published:** 2019-11-04

**Authors:** Vincent Trung H Ngo, Homero Camacho, Jasbir Singh

**Affiliations:** 1 Department of Psychiatry, UCLA-Kern, Ross University School of Medicine, Bakersfield, USA; 2 Department of Psychiatry, UCLA-Kern, Kern Medical, Bakersfield, USA

**Keywords:** postictal psychosis, epilepsy, psychosis, postictal, schizophrenia, eeg, psychosis of epilepsy, ictal, interictal, epilepsy seizures

## Abstract

Postictal psychosis is a rare but potentially serious complication in patients with seizure disorders. There is no consensus on best practices in managing and treating postictal psychosis as well as other psychoses of epilepsy, but current research is investigating topics such as adherence to seizure medications and antipsychotic administration during or before psychosis and addressing psychosocial stressors as potential components of effective treatment. We present a case report detailing a patient’s lengthy history of postictal psychosis due to her underlying temporal lobe epilepsy; her disease course as correlated by a diagnostic electroencephalogram (EEG), her history of medication nonadherence, and the treatment of postictal psychosis are also discussed.

## Introduction

The observation of psychotic symptoms in a patient can be alarming and adds a layer of complexity to a patient’s diagnostic history as well as management. Our patient, who began having seizures at a young age, encountered us when suspicion for psychotic symptoms arose. Postictal psychosis (PIP) is part of a group of psychotic disorders known as psychosis of epilepsy (POE), which are believed to either originate from or are pathogenically related to a seizure disorder. POE also includes interictal psychosis (IIP), which is distinct semiologically from a primary schizophrenic disorder, as well as ictal episodes [1]. Logsdail et al. determined that a clear temporal relation existed in PIP between the bout of partial or generalized seizure and the subsequent psychotic state, and created a list of criteria that continues to be used for the diagnosis of PIP: (1) episode of psychosis within one week after a seizure(s); (2) the psychosis lasts more than 15 hours and less than two months; (3) delusions, hallucinations in clear consciousness, bizarre or disorganized behavior, formal thought disorder, or affective changes; and (4) no evidence of antiepileptic drug toxicity, non-convulsive status epilepticus, recent head trauma, alcohol, drug intoxication, withdrawal, or prior chronic psychotic disorder [2]. The Diagnostic and Statistical Manual of Mental Disorders, 5th Edition (DSM-V) has placed PIP in the category of “psychotic disorder due to another medical condition” and has kept these criteria. The etiology and pathology of POE have yet to be established but associations with localization-related epilepsy, particularly temporal lobe epilepsy, have been found [3]. PIP progression to IIP has also been observed, with a transition period between months to years [4-5], although they still represent distinct clinical entities with potentially different neurobiological backgrounds [3]. IIP, unlike PIP, is a psychosis not temporally related to a seizure in a patient with previously diagnosed epilepsy [5].

Epidemiological studies have been revealing in terms of the prevalence of epilepsy, with inconsistent findings on risk or frequency of psychosis in epilepsy. For example, population-based studies have estimated the lifetime prevalence of epilepsy at 1.2% of all adults in England [6], and a three-fold increased risk of schizophrenia-like psychosis was found in patients with epilepsy (PWE) in Denmark [7]. In a systematic review, PIP had a frequency of 4% among all reported postictal manifestations in PWE [8]. An almost eight-fold risk of psychosis in PWE was found in another systematic review, with a pooled estimate of the prevalence of psychosis in epilepsy at 5.6% (temporal lobe epilepsy psychosis at 7%, interictal psychosis at 5.2%, postictal psychosis at 2%) [9]. Possible risk factors for POE found in the literature include a family history of epilepsy, a family history of psychosis, a family history of mood disorders, temporal lobe and other partial epilepsies, bilateral or widespread central nervous system (CNS) injury, EEG slowing, and borderline intelligence [10].

There is no current consensus on best practices for the management and treatment of PIP, which often makes providing appropriate care to patients challenging. Further complicating the discussion on PIP management is the widely accepted idea that antipsychotic medications, typically used to treat psychosis, can lower the seizure threshold. Nonetheless, the use of antipsychotics in the treatment and management of PIP remains a popular topic of research. Some clinicians recommend antipsychotics and benzodiazepines for symptomatic treatment for PIP and brief IIP [11]. Current proposals for approaches to the management of PIP and IIP advised accurate and early diagnosis, the optimization of seizure medication, the initiation of antipsychotic medication, and the promotion of basic psychosocial interventions to enhance adherence [11].

We present a patient with temporal lobe epilepsy who, over the span of several years, presented with numerous episodes of postictal psychosis. We postulate that a likely precipitating factor of her postictal psychosis is the increased frequency of seizures due to medication nonadherence.

## Case presentation

This is a 30-year-old Hispanic female with a history of mild intellectual delay and temporal lobe epilepsy with postictal psychosis complicated by poor medication adherence. She has an extensive chart history in the hospital system, with numerous admissions in the emergency, internal medicine, obstetrics/gynecology, and psychiatry departments. Her earliest documented EEG in the hospital chart at 22 years of age showed spikes with phase reversing in the left and right temporal regions, indicative of an underlying structural abnormality, with moderate generalized slowing. She came to our attention in the department of psychiatry as a consult request for the evaluation of psychosis, also at the age of 22. She was admitted for birth out of asepsis, having given birth at home, and brought to the hospital for further care, whereupon a consult was placed for the reported presence of psychotic symptoms. As she did not meet the criteria for any psychiatric disorders at the time, psychiatry deemed her symptoms a manifestation of ictal episodes due to her temporal lobe epilepsy. Collateral information from the patient’s mother revealed that she began having seizures at 13 years of age, with increasing frequency of seizures during her pregnancy. The patient’s mother also noted that she was poorly adherent to her then-current antiepileptic medications of valproic acid 250 mg and oxcarbazepine 600 mg. From the information given by the mother, it was unclear whether or not this regimen changed for the patient during pregnancy.

The psychiatry team encountered the patient two years later, the patient was brought to the emergency department on a 72-hour involuntary psychiatric hold for danger to self and danger to others. She was found to be walking in the middle of oncoming traffic and was seen attempting to kidnap a child from a gas station, mistaking her as her own. The patient was discovered to have been nonadherent with her antiseizure medications in the interim, with increasing frequency of seizures occurring, and had been seen in the emergency department four days prior for a seizure with fall, with right facial trauma. Medications per chart were levetiracetam 1000 mg BID, and valproic acid 250 mg QHS. On evaluation, the patient was disorganized, confused, and paranoid, making accusatory statements to the nursing staff, “you are the one that was beating me, even though my husband had asked you to stop, you continued to beat me and you brought me here.” In regards to the attempted kidnapping, the patient stated she thought she was taking “her own daughter.” She stated that she had been depressed since delivering her most recent child last month (per chart, she had a normal spontaneous vaginal delivery recorded three months prior) and because she lost all her children (per chart, children were in custody of child protective services). She was started on valproic acid 250 mg BID and diazepam 2 mg BID for seizure prophylaxis; levetiracetam was held at this time due to the possibility of worsening psychosis and depression. During her psychiatric hospitalization, the patient suffered a witnessed tonic-clonic seizure lasting for at least a minute and a half after screaming and falling out of a chair; subsequent facial and cranial X-rays showed no fractures or apparent injury. According to the Diagnostic and Statistical Manual of Mental Disorders Four (DSM-IV) criteria, the patient was given an Axis I diagnosis of psychosis not otherwise specified (NOS), with ruling out of postpartum depression with psychosis. The patient was transferred to the medicine floor for the intravenous (IV) administration of antiseizure medication and further observation. EEG showed multiple sharp phases reversing in the left frontotemporal area, less frequently in the right temporal area, with moderate slowing. Valproic acid was increased to 500 mg BID per neurology recommendations. The patient was ultimately discharged with a diagnosis of psychosis NOS, with additional ruling out of postictal psychosis and postpartum depression with psychotic features.

Five months later, the patient was again seen by psychiatry while in the emergency department for an involuntary psychiatric hold evaluation due to reports of the patient attempting to pick up children that did not belong to her from a school. The patient at this time was found to have a subtherapeutic valproic acid level of 40, was selectively mute, and was pregnant with unknown gestational age. She was admitted inpatient for the management of her seizures and the resolution of her altered mental state. She reported delusions of “children in the room, seven of them” upon evaluation by the psychiatry consultation team. Prior to discharge, she was restarted on oxcarbazepine 600 mg BID, and valproic acid was decreased from 500 mg BID to 250 mg BID for one week and then discontinued.

Three years later, at the age of 27, the patient saw an outpatient neurologist for the evaluation of her refractory epilepsy. She was recently switched to levetiracetam 1000 mg BID from valproic acid 500 mg BID but continued having one to three seizures a week, with intermittent seizure clusters in a day. Follow-up visits the next month showed a worsening of seizures to twice a day; levetiracetam was increased to 1500 mg BID and a one-day course of phenytoin 100 mg TID was given.

The psychiatry team was reconsulted two years after this (at the age of 29) after the patient was transferred to the emergency department from another hospital with seizure with altered mental status, presenting with disorientation and flight of ideas. The patient was pregnant during this encounter at nine weeks gestation. On the initial visit, the patient was disorganized and provided with haloperidol 2.5 mg intramuscular and diphenhydramine 25 mg IV for agitation. At a later encounter, the patient was more cooperative but still disoriented, endorsing voices in the room of “10 people that talk about everything and can talk about her.” Collateral information from her mother at this time revealed that the patient typically becomes altered after a seizure and has presented in a similar manner in the past, with courses lasting 15 days to one month after experiencing a seizure. Considering the new collateral information, the patient was given the diagnosis of temporal lobe epilepsy with postictal psychosis upon this admission.

**Figure 1:**
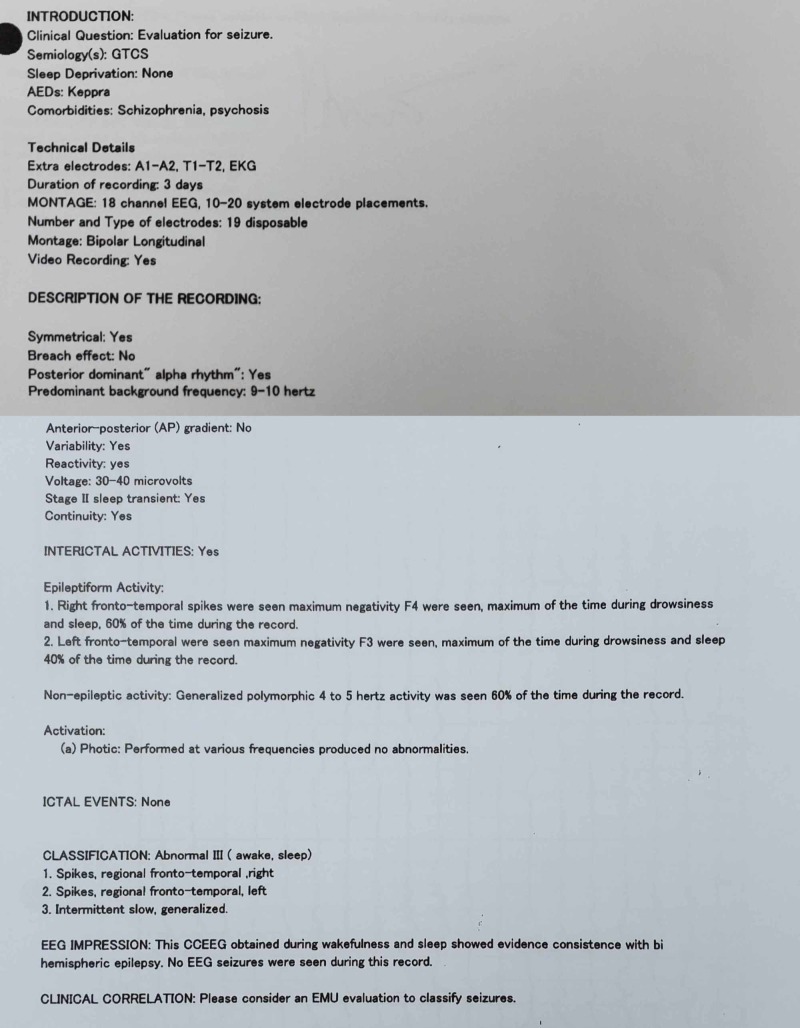
EEG results and interpretation Please note that the comorbidities listed were part of the working differential for the patient.

**Figure 2:**
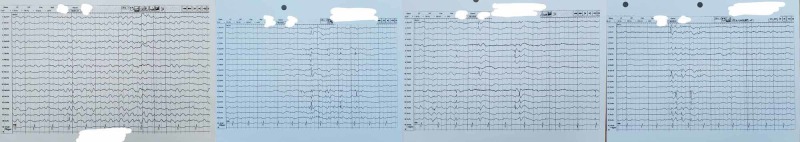
A 48-72 hour EEG reading from hour 12, approximately 30-minute window

**Figure 3:**
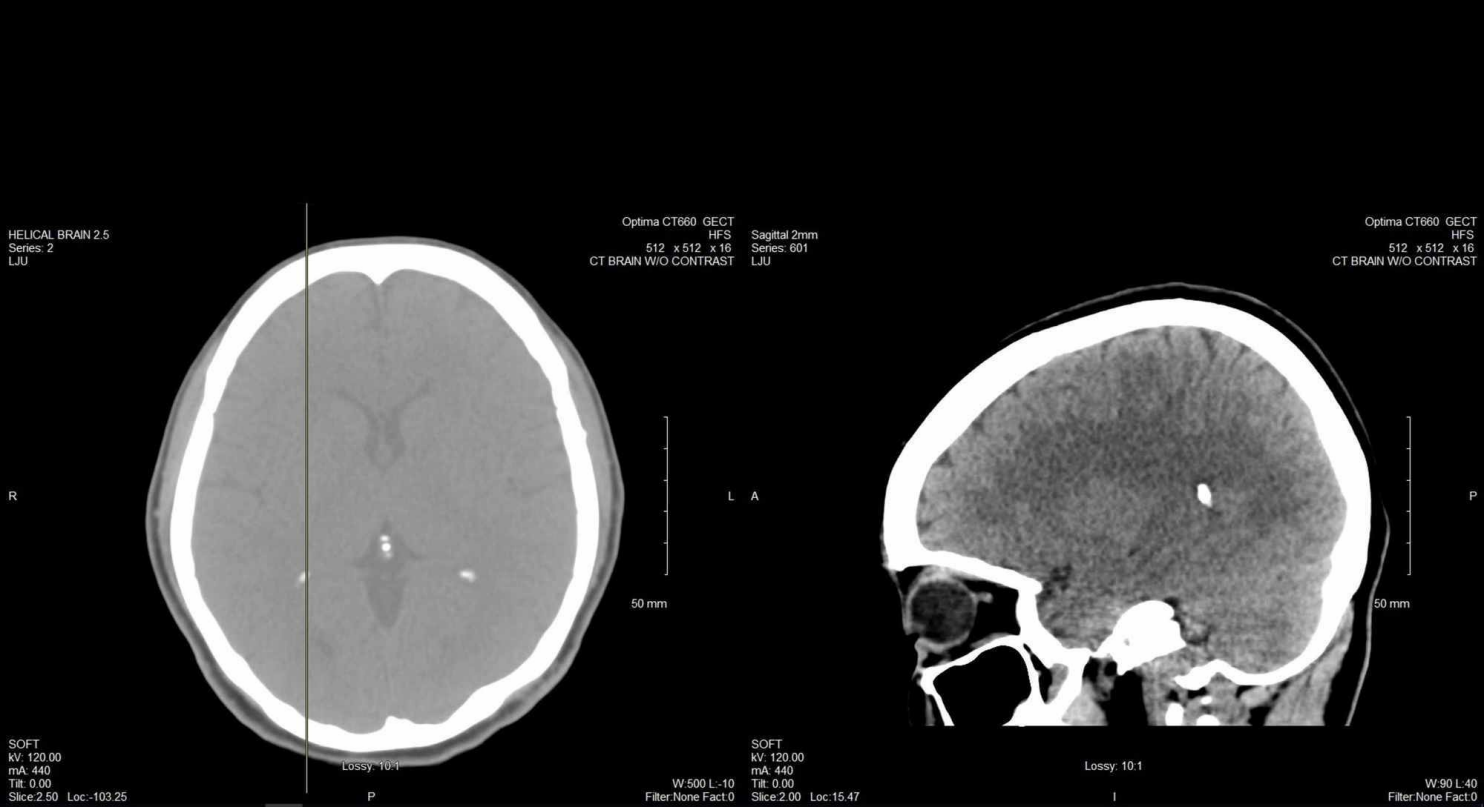
Axial and sagittal views of CT brain without contrast, highlighting the periventricular white matter hypodensities.

**Figure 4:**
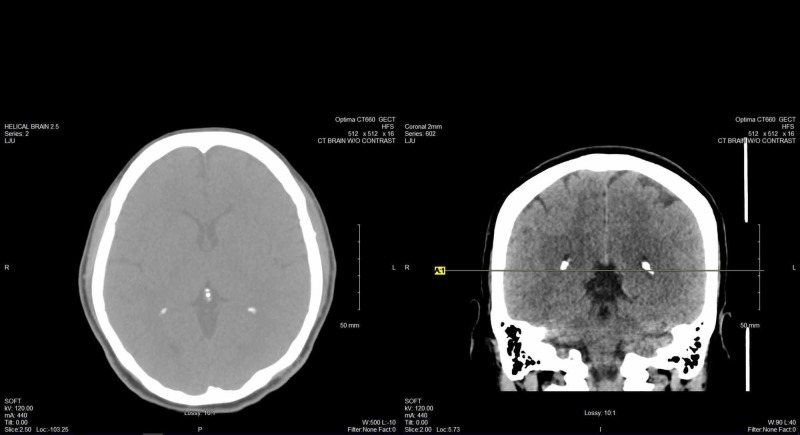
Axial and coronal views of CT brain without contrast, highlighting the periventricular white matter hypodensities CT: computed tomography

During the next month, the patient was once again seen by the psychiatry team. She was taken to the emergency department by the ambulance after being found trying to break into another person’s home. When speaking to law enforcement at the scene, the patient stated that she was trying to enter her own home to get her children but could not provide context for the situation. She was also displaying “strange behavior” per the chart and thus was taken to the emergency department for further evaluation. The patient was currently pregnant at 15 weeks at this admission and found to have been noncompliant with her seizure medications. levetiracetam 500 mg BID was started while inpatient, and a 48-72 hour video EEG was recommended for the patient per neurology (Figures 1-2). Although postictal psychosis was discussed on previous admissions, efforts were made to rule out psychosis unrelated to seizure activity with computed tomography (CT) brain without contrast; neuroimaging results showed no acute or structural abnormalities with the exception of hypodensities in the periventricular white matter region (Figures 3-4). Lacosamide 150 mg IV Q12H was started and levetiracetam discontinued. Further psychiatric evaluation ruled out the possibility of a psychiatric cause of the patient’s current presentation.

Since then, the patient has continued to have repeated encounters in the hospital system, twice for episodes of seizures with no presentation of psychoses in her postictal state. This is typical of the patient’s overall history, as she has had numerous emergency department visits since the age of 24 for either seizure episodes or seizure medication refills. The current medication on record for the patient is levetiracetam 1000 mg BID.

## Discussion

PIP is a serious but possibly preventable complication of epilepsy, affecting over 2% of the patient population [9]. The relationship between epilepsy and POE is bidirectional, as PWE are at greater risk of developing a psychotic disorder and vice versa. This may be due to structural brain abnormalities, such as cortical dysgenesis or diffuse brain lesions, being involved in the pathogenesis of both disorders. Seizures have also been shown to modify the presentation of psychosis [12]. Thus, cases of POE must rule out comorbid psychiatric disorders as well as medication-induced psychosis before concluding a diagnosis [1]. In our patient’s case, her longstanding history of temporal lobe epilepsy (unclear age of onset), confirmed by EEG findings of left and right temporal spike with phase reversing, was indicative of an underlying structural abnormality in this region. Collateral information was critical for narrowing the diagnostic differential to postictal psychosis. The patient’s overall treatment was ultimately hampered by medication nonadherence and loss to follow-up. Potential novel aspects of this case report are the lack of targeted treatment of PIP and the patient’s gravida/parity. We will also look at EEG findings over the course of this patient’s disease and discuss implications for diagnosis.

Evidence-based treatment modalities are not currently established for PIP or IIP, but there are current recommendations based on literature reviews and expert opinions. PIP treatment recommendation consists of acute protective measures and preventive procedures. Family education is an important component of management; once PIP develops fully from nascent behavioral changes after seizures, protective custody or involuntary holds may be necessary to prevent the patient’s harm to self or others. In our patient’s case, this aspect of care may have helped prevent her erratic behavior involving walking out into traffic, attempting to abduct children, and attempting to enter someone else’s residence. Treatment with medication in PIP is more conservative with the use of antipsychotics than in IIP, which advocates for low-dose antipsychotic use at the first signs of symptoms. Patients with PIP have psychosis only during the postictal phase, unlike IIP, and the optimization of a patient’s antiseizure medication to stop seizures will subsequently prevent PIP as well. A related adverse effect of antiseizure medications is the risk of psychosis after administration; a retrospective study showed that psychosis after administration was related to the type of seizure medication, specifically lamotrigine and topiramate, rather than dosage [11]. Medication options during the acute phases of PIP depend on the severity of the psychosis: low-dose benzodiazepines for patients without alarming behaviors, combined benzodiazepine and antipsychotic for agitated or hyperexcitable psychoses, and intramuscular antipsychotics for noncompliant patients or patients with a past history of violent episodes. The continuous use of antipsychotics and benzodiazepines are generally not recommended for use outside of the psychotic episodes [11]. In addition, other clinicians advocate claims that low doses of antipsychotics when coupled with an effective antiseizure medication regimen, will not significantly elevate the risk of inducing a seizure in PIP patients and PWE [11]. In terms of antipsychotic efficacy, there is currently insufficient data on the relative efficacy of any one drug over another to inform practice; a literature review of drug randomized controlled trials (RCTs) showed only one study comparing the superior efficacy of olanzapine over haloperidol in the treatment of a small population of patients with POE [13]. Electroconvulsive therapy and repetitive transcranial magnetic stimulation have also been postulated to aid in PIP treatment [11]. Lastly, surgery for epilepsy can lead to the successful treatment of PIP, particularly dominant temporal lobectomies but may place patients at elevated risks for subsequent mood disorders [14].

EEG is an important diagnostic modality in evaluating patients with epilepsy and usually serves as an adjunct tool in confirming the diagnosis or identifying subtypes. EEG abnormalities have been known to persist during psychotic episodes in PIP, with the studies showing a diverse but also inconsistent range of EEG findings. Early studies suggested a higher prevalence of temporal lobe involvement in epilepsy patients who experienced schizophreniform psychosis [12], with associations made between PIP and resistant temporal lobe epilepsy stemming from mesial temporal sclerosis, particularly on the left side [15]. Other studies have observed higher rates of ictal fear, bilateral independent discharges, and gross structural lesions in PIP [16] while additional studies show no differentiating EEG findings between psychotic and nonpsychotic PWE [17]. Yet another case study has shown a patient with right temporal lobe epilepsy undergoing a hyper-religious psychotic experience, with increased activity in the left prefrontal cortex suggesting a possible mechanism related to prefrontal lobe processes rather than medial temporal-lobe processes [18].

Considering the diversity of EEG findings, the pathogenesis of PIP is not well understood. Some of the purported mechanisms or phenomena related to PIP pathogenesis include the involvement of ictal activity in the temporal lobe, suggested by frequent subictal discharges in that region; changes in postsynaptic dopamine receptor sensitivity; and neurochemical changes such as increased turnover of gamma-aminobutyric acid (GABA) or reductions in the cerebral concentrations of aspartate and glutamate [12]; reduction of bilateral posterior hippocampal volumes in POE [19]; and postictal hyperperfusion, particularly in the bifrontal and bitemporal regions [20].

In our patient, EEG findings spanned the course of her hospital chart, with the earliest EEG done at the age of 22 during a state of clear consciousness, showing left spike phase reversing in the left and right temporal areas, left greater than right, with the right temporal spike indicating an underlying structural abnormality. A moderate generalized slowing was also shown, presumably deemed to be due to sedation versus encephalopathy. The patient’s next EEGs were at the age of 24 during admission for an apparent psychotic episode. The EEG for this period was during a postictal state and showed multiple spikes and sharp waves phase reversing in the left frontotemporal area and right temporal area, occurring independently. Another EEG during that admission was done during a period of psychosis and showed independent sharps in the left and right temporal areas but was shortened due to the patient's uncooperativeness. An EEG that was done at the age of 26 while the patient was in active labor, and postictal per family, showed no epileptiform discharges, with intermittent generalized slowing. The most recent EEG at the age of 29 was done during a period of psychosis and showed left and right frontotemporal spikes with intermittent generalized slowing. These findings correlate with studies previously discussed showing no differences in EEG findings between psychotic and nonpsychotic PWE. A progression of EEG abnormalities accumulated over the years, from bilateral temporal to bilateral frontotemporal, cannot be ruled out. Also, it is unknown if there were similar psychotic episodes post-seizure for which the patient did not have an encounter in the hospital system; considering the span of time that the patient’s history encompasses, this limiting factor also cannot be ruled out.

We believe this case report encompasses many considerations, concerning the diagnosis and treatment of PIP. Firstly, our patient was pregnant on two separate hospital admissions where a postictal psychosis was observed. Though we know that her seizures increased in frequency during pregnancy, to what extent, if any, the pregnancy affected the patient’s psychosis in terms of etiology is unclear. Indeed, the characterization of the patient's disease process may have been bolstered by a more detailed history; clinicians are more reliant on history when treating diseases with poorly understood pathology such as postictal psychosis. Also, the patient’s EEG results throughout her disease were indicative of an underlying structural abnormality as the cause of her epilepsy, but no discernible unique abnormalities were found during her states of psychosis. The presence of EEG changes directly caused by the psychosis, however, still cannot be ruled out. Furthermore, antipsychotics and benzodiazepines were used to treat the patient’s psychotic symptoms on at least two occasions but were not used as targeted treatment against her PIP per current literature recommendations. Lastly, the patient’s numerous emergency department visits for medication refills and seizure episodes present a unique challenge in creating effective behavioral or social interventions to address the unnecessary use of healthcare resources, poor medication adherence, and overall quality of care. POE and PIP are disorders with largely unknown pathogenic mechanisms as well as no current guidelines for treatment, but there is still much potential for the expansion of our knowledge in this area.

## Conclusions

In conclusion, this case report adds to the body of knowledge regarding the characterization of the disease process of postical psychosis, which may add to the current discourse regarding management and treatment modalities. Also, further clinical research is needed to discover more about the pathology behind the disease.
